# In Myotonic Dystrophy Type 1 Head Repositioning Errors Suggest Impaired Cervical Proprioception

**DOI:** 10.3390/jcm13164685

**Published:** 2024-08-09

**Authors:** Stefano Scarano, Antonio Caronni, Elena Carraro, Carola Rita Ferrari Aggradi, Viviana Rota, Chiara Malloggi, Luigi Tesio, Valeria Ada Sansone

**Affiliations:** 1 Department of Biomedical Sciences for Health, University of Milan, 20133 Milan, Italy; s.scarano@auxologico.it (S.S.); valeria.sansone@centrocliniconemo.it (V.A.S.); 2 IRCCS Istituto Auxologico Italiano, Department of Neurorehabilitation Sciences, Ospedale San Luca, 20122 Milan, Italy; v.rota@auxologico.it (V.R.); chiaradiletta.malloggi@ospedaleniguarda.it (C.M.); l.tesio@auxologico.it (L.T.); 3 The NeMO Clinical Center in Milan, Neurorehabilitation Unit, University of Milan, 20162 Milan, Italy; elena.carraro@centrocliniconemo.it (E.C.); carola.ferrari@unimi.it (C.R.F.A.)

**Keywords:** myotonic dystrophy, muscle spindle, ataxia, balance, posturography, cervical proprioception, joint position error, optoelectronic

## Abstract

**Background:** Myotonic dystrophy type 1 (DM1) is a rare multisystemic genetic disorder with motor hallmarks of myotonia, muscle weakness and wasting. DM1 patients have an increased risk of falling of multifactorial origin, and proprioceptive and vestibular deficits can contribute to this risk. Abnormalities of muscle spindles in DM1 have been known for years. This observational cross-sectional study was based on the hypothesis of impaired cervical proprioception caused by alterations in the neck spindles. **Methods:** Head position sense was measured in 16 DM1 patients and 16 age- and gender-matched controls. A head-to-target repositioning test was requested from blindfolded participants. Their head was passively rotated approximately 30° leftward or rightward and flexed or extended approximately 25°. Participants had to replicate the imposed positions. An optoelectronic system was adopted to measure the angular differences between the reproduced and the imposed positions (joint position error, JPE, °) concerning the intended (sagittal, horizontal) and unintended (including the frontal) planar projections. In DM1 patients, JPEs were correlated with clinical and balance measures. Static balance in DM1 patients was assessed through dynamic posturography. **Results:** The accuracy and precision of head repositioning in the intended sagittal and horizontal error components did not differ between DM1 and controls. On the contrary, DM1 patients showed unintended side-bending to the left and the right: the mean [95%CI] of frontal JPE was −1.29° [−1.99°, −0.60°] for left rotation and 0.98° [0.28°, 1.67°] for right rotation. The frontal JPE of controls did not differ significantly from 0° (left rotation: 0.17° [−0.53°, 0.87°]; right rotation: −0.22° [−0.91°, 0.48°]). Frontal JPE differed between left and right rotation trials (*p* < 0.001) only in DM1 patients. No correlation was found between JPEs and measures from dynamic posturography and clinical scales. **Conclusions:** Lateral head bending associated with head rotation may reflect a latent impairment of neck proprioception in DM1 patients.

## 1. Introduction

Myotonic dystrophy type 1 (DM1) is a rare autosomal dominant genetic disorder with a prevalence estimate of 5 to 20 per 100,000 individuals [1,2,3]. DM1 represents one of the most common muscular dystrophies in adults worldwide [4]. It is a multisystemic disease caused by a repeat expansion of the CTG trinucleotide in the dystrophy myotonic protein kinase (DMPK) gene [5]. DM1 presents various signs and symptoms, including muscle weakness, myotonia, early-onset cataracts, cardiac arrhythmias, cognitive impairments, and central and peripheral nervous system dysfunctions [6]. The cardinal feature of DM1 is myotonic myopathy, consisting of myotonia (difficulty in relaxing muscles after contraction) [7], weakness, and muscle wasting with preferential involvement of facial, trunk, and distal limb muscles. The muscle involvement in DM1 shows features common to other neuromuscular disorders (from myasthenia to limb–girdle myodystrophies): neck flexor muscles are affected early in the disease progression, and the motor impairment of the limbs progresses from distal to proximal [4,8].

DM1 patients show impairments of balance and gait [9,10,11,12,13], with a 10-fold increase in the risk of falling compared to age-matched healthy controls [9]. Traditionally, muscle weakness in the distal and proximal lower limbs, myotonia, cognitive impairment, and daytime sleepiness have been considered the main risk factors for falling in these patients [14,15]. However, previous research has highlighted that DM1 patients are affected by somatosensory [16,17] and audio-vestibular [18,19] abnormalities. Furthermore, a recent study has revealed that both the proprioceptive and vestibular systems can be involved [20].

Multiple sensory inputs, mainly provided by the visual, vestibular, and somatosensory afferents, must be integrated to keep balance [21,22]. By somatosensory, we refer to those inputs also leading to proprioception, i.e., the sensation of joint position and movement [23,24]. Proprioception (also referred to as kinesthesia and named initially “muscle sense” [25]) involves signals from mechanoreceptors located within muscles, skin, tendons, and joints and requires their integration [26]. Among mechanoreceptors, muscle spindles play a central role in proprioception [27] and they are currently considered the main kinesthetic sensors [24]. Spindles are receptors consisting of nerve endings and specialized muscle fibers arranged in parallel with the other muscle fibers and surrounded by connective tissue. These structures act as stretch-sensitive transducers [28], informing the nervous system about the changes in the length of muscles and the speed and acceleration of such changes [24,29,30]. Nearly every striated muscle—except facial ones and some sphincter muscles [31]—contains muscle spindles [30], but these are particularly abundant in extraocular, hand and—of interest here—neck muscles [32,33].

As anticipated, the main sensory inputs underpinning balance control are provided by the vestibular, visual and proprioceptive systems [21]. In addition, cutaneous afferents from the soles of the feet contribute to the lower limbs’ proprioceptive cues and the sense of body movement with respect to the ground [34].

Proprioceptive information from the cervical spine is integrated with vestibular and visual feedback to control both the head position and whole-body posture [35]. Also, information from the neck proprioceptors signaling the position and motion of the head relative to the trunk is considered necessary for the proper functionality of the vestibular system [36,37]. The skeletal muscles of DM1 patients show distinctive histological alterations [38], such as the progressive replacement of muscle tissue by fat and fibrous tissue and necrotic muscle fibers [39]. Pronounced abnormalities also affect the muscle spindles of these patients [30,39,40,41]. The spindle fibers appear abnormally thin because of longitudinal splitting and fragmentation [40]. Also, alterations of spindle motor and sensory innervation have been described [41,42].

Previous research has investigated the ultrastructural changes in muscle spindles in DM1 patients [30]. To the authors’ knowledge, no prior studies on DM1 patients investigated joint proprioception in vivo. Some studies have investigated the presence of peripheral sensory neuropathy in these patients, although with mixed results [43].

Head motion and vestibular functionality are strictly related [44]. Considering the afferent and efferent connections of the cervical muscles with the visual and vestibular apparatus [45], it can be hypothesized that an impairment of the neck muscle spindles could manifest as a vestibular impairment of balance. With this rationale, the present study aims to measure neck proprioception in patients with DM1 and correlate these data with balance measures and clinical assessments. This study would thus add clinical significance to neck proprioception impairment and suggest new potential targets for therapeutic exercise aimed at improving balance.

## 2. Materials and Methods

This observational cross-sectional study (ClinicalTrials.gov: NCT04712422) complied with the Declaration of Helsinki and was approved by the ethical committee of the IRCCS Istituto Auxologico Italiano (CABLAMYD project, Ricerca Corrente IRCCS).

### 2.1. Participants

From October 2020 to September 2021, 16 DM1 patients and 16 healthy controls were recruited. All participants gave their informed consent to participate in the research.

The sample of DM1 patients recruited in the present study is the same as that enrolled in a previous study from the authors’ research group, which aimed to assess and characterize the impairment of standing balance in DM1 [20].

DM1 patients were recruited according to the following criteria:

Inclusion criteria:

1. Genetically confirmed patients with DM1, classified according to the number of CTG repeats: E1 (CTG repeats: 50–150), E2 (150–1000), and E3 (>1000) [46].
2. Age between 18 and 50 years.
3. Ability to keep upright without assistance or assistive devices for at least 20 s.
4. Rivermead Mobility Index [47] score ≥ 10/15.
5. Visual acuity > 10/20 (corrective lenses allowed).
6. Mini-Mental State Examination [48] score ≥ 26/30.

Exclusion criteria:

1. Any balance impairment caused by a neurological or cardiovascular disease, musculoskeletal disorder, or other pathological conditions suspected to affect the results of the tests to be performed.
2. Pregnancy.
3. Any previous major orthopedic surgery.
4. Head or neck trauma in the six months preceding this study.

DM1 patients were recruited among the NeuroMuscular Omniservice Clinical Center (NEMO) outpatients in Milan, Italy, a dedicated Clinical Center for neuromuscular diseases.

A sample of age- and gender-matched healthy controls were recruited, within the same age range and complying with exclusion criteria. Healthy controls were recruited among the personnel and the visitors of the Department of Neurorehabilitation Sciences, IRCCS Istituto Auxologico Italiano in Milan, Italy.

DM1 patients received a neurologic assessment and an instrumental assessment of balance and cervical proprioception. Controls only received the instrumental evaluation of cervical proprioception. All patients were naïve to the instrumental balance assessment used in this study. All participants were naïve to cervical proprioception tests.

A neurologist expert in DM1 performed the clinical assessment at the NEMO Centre. A physiatrist and a bioengineer instrumentally evaluated balance and cervical proprioception at the Department of Neurorehabilitation Sciences of the IRCCS Istituto Auxologico Italiano in Milan. The clinical and instrumental assessments occurred on the same day for each DM1 patient.

### 2.2. Clinical Assessment

The clinical assessment was performed on the DM1 patients only.

Clinical and demographic data were recorded. As described in detail in [20], in the DM1 patients, the severity of muscle impairment, the mobility in balance and transfers, and the perception of dizziness were assessed through rating scales.

The Muscular Impairment Rating Scale (MIRS) [49] was used to measure muscular impairment in DM1, as it was conceived to reflect the distal to proximal progression of the muscular involvement in DM1. The MIRS consists of an ordinal five-point rating scale and is partly based on manual muscle testing of 11 muscle groups: the neck flexors, six proximal muscle groups (shoulder abductors, elbow flexors, elbow extensors, hip flexors, knee extensors, knee flexors), and four distal muscle groups (wrist extensors, digits flexors, ankle dorsal flexors, and ankle plantar flexors). Of note, a score of 1 stands for no muscular impairment, 3 for distal weakness, and 5 for severe distal and proximal weakness.

The Rivermead Mobility Index (RMI) [47] was adopted to quantify patients’ independence in mobility. The RMI is a 15-item scale. Each item can be scored 1 or 0, based on the patient’s capacity to perform each activity independently or not. Higher RMI total scores stand for greater independence in mobility.

The Dizziness Handicap Inventory—short form (DHIsf) [50] is a 13-item scale measuring the severity of self-reported balance deficits. The DHIsf was developed through Rasch Analysis [51,52] from the original 25-item Dizziness Handicap Inventory scale. The total score ranges from 0 to 13, with higher scores indicating better balance.

The number of falls in the previous 12 months was retrospectively recorded.

### 2.3. Cervical Proprioception Instrumental Assessment

The instrumental assessment of cervical proprioception was performed on DM1 patients and healthy controls.

Different tests have been developed for assessing proprioception [29]. This study used a joint position reproduction test [29]. Full details of the procedure are reported in our previous work [53].

In brief, the evaluation involved a head-to-target (HTT) repositioning test [54]. An optoelectronic procedure (see below) was utilized to capture the head position. During the HTT repositioning test, an operator gently guides a blindfolded participant’s head from the starting head position to a target position. Next, the participant returns to the starting position and is asked to actively reproduce the position previously imposed by the operator. The angular difference between the position autonomously produced by the participant and the one imposed by the operator was defined as joint position error (JPE) and calculated in degrees [29]. The JPE is used as a measure of proprioception, with a greater JPE (i.e., an increased angular difference) standing for worse proprioception. Therefore, each trial in the current HTT repositioning test comprised an operator-assisted head motion followed by an autonomous head motion. For each trial, the positions achieved at the end of these two motions were compared to calculate the JPE.

At the beginning of the procedure, the starting head position was identified for each participant. The participant was requested to assume a comfortable sitting posture on a chair with back support and armrests. Then, they were asked to stick to the backrest and look straight ahead. This head position was defined as the starting head position. The sitting position was maintained for the entire duration of the assessment.

The target positions consisted of 30° right or left head rotations on the horizontal plane and 25° flexion or extension on the sagittal plane. Unavoidably, the operators’ and participants’ positions could not precisely match the intended range or be strictly confined to the intended planes [53]. Each of the four directions was tested four times (i.e., four trials per direction), so each participant performed sixteen trials. The order of trials was quasi-randomized (see the Supplementary Materials from [53] for details on the trials’ quasi-randomization). As anticipated, the participants were blindfolded for the procedure and were not provided with any information about their performance.

The motion capture procedure used here has been detailed in our previous study [53]. Participants were equipped with ten spherical self-adhesive reflective markers, 1 cm in diameter. Seven markers were placed on a rubber “crown” on the participant’s head, while three markers were applied to the skin covering the upper trunk (sternum, left and right acromion). Eight near-infrared stroboscopic cameras (Smart-D optoelectronic system; BTS™ Bioengineering SpA, Milan, Italy, sampling rate 100 Hz) were used to capture the 3D displacement of the markers. Each raw marker’s signal was interpolated through a cubic spline curve and smoothed through a triangular window bandpass filter. The optoelectronic system enabled the JPE to be broken into three orthogonal planes.

Signal analysis was conducted in SMART-Analyzer through a customized protocol (BTS Bioengineering Spa, Milan, Italy).

As mentioned above, head-to-target motions are characterized by “unintended” movements in secondary planes, i.e., in planes different from the one of the requested motion [55] (e.g., participants may move their head in the frontal plane when only left or right head rotations in the horizontal plane are requested). Strictly speaking, the adjectives “intended” and “unintended” apply to the rotations imposed by the operator. The patient’s task was replicating the imposed rotation, whichever its orientation in space. For simplicity, these terms were applied here to both the passive and the active movements.

For each trial, three JPEs were calculated: JPE*horizontal*, JPE*frontal*, and JPE*sagittal*. These three JPEs consisted of the error components projected in the horizontal, frontal, and sagittal planes.

Among these three JPEs, for each type of trial (i.e., flexion, extension, right rotation or left rotation), an intended JPE component (JPE*int-component*) and two unintended JPE components were identified. The JPE*int-component* consisted of the JPE in the plane of the intended movement. The JPE*int-component* corresponded to JPE*horizontal* in trials testing right and left rotations. In contrast, it corresponded to JPE*sagittal* in trials testing flexion and extension.

The other two JPEs were unintended JPEs: JPE*horizontal* and JPE*frontal* in the trials of flexion and extension, and JPE*sagittal* and JPE*frontal* in the trials of right and left rotations.

As no trial tested repositioning in the frontal plane (i.e., side-bending), JPE*frontal* was always an unintended component.

It is worth reminding that the “error” in repositioning arises from the difference between the angles achieved passively (i.e., at the end of the operator-assisted head motion) and actively (i.e., at the end of the following autonomous head motion), respectively, in the same trial.

To note, in our previous paper [53], the error was also computed for the operator with respect to the requested angular positions (i.e., 30° to the right or the left for trials on the horizontal plane and 25° flexion or extension for trials on the sagittal plane) and found to be minimal.

The JPE*int-component* can be positive (indicating an overshoot of the target position) or negative (indicating an undershoot). Unintended JPEs can be positive or negative as well. Positive JPE*sagittal* or JPE*horizontal* values indicate rotation to the right or extension, respectively. Negative JPE*sagittal* and JPE*horizontal* correspond to rotation to the left and flexion, respectively. A positive JPE*frontal* indicates side-bending to the right in the frontal plane (i.e., clockwise from the examiner’s perspective). At the same time, a negative JPE*frontal* implies side-bending to the left (i.e., counterclockwise from the examiner’s perspective). For further details on JPEs definition and calculation, see [53].

The JPE*3D* was also calculated as a complementary analysis (see [53], and Supplementary Materials). This value is the angle between the head longitudinal axis at the end of the passive and the active movements, whichever the orientation of this angle. JPE*3D* can only be zero or positive.

### 2.4. Balance Instrumental Assessment

The balance instrumental assessment was performed on DM1 patients only. As already recalled, full details on the balance instrumental assessment of this same sample of DM1 patients are reported in a previous study [20]. In short, for the standing balance assessment, a computerized posturography device was adopted (EquiTest^©^, Neurocom International Inc., Clackamas, OR, USA). The EquiTest instrument consists of a dual-force plate, which can rotate in the sagittal plane, and a mobile visual surround, depicting a stylized landscape encircling the participant. The sensory organization test (SOT) of the EquiTest system measures the anterior-posterior displacement of the participant’s center of mass (CoM), estimated from the center of pressure recorded by the force plates, during different standing conditions. As anticipated, the visual surround and the support may move, separately or together, matching the sways of the participant’s CoM (i.e., sway-referenced). In this way, the EquiTest SOT protocol stresses the patient’s ability to effectively integrate visual, vestibular and proprioceptive inputs with muscular recruitment while standing.

Detailed descriptions of the EquiTest SOT are available elsewhere [22,56]. Six balance conditions were tested: condition 1 = eyes open, firm support; condition 2 = eyes closed, firm support; condition 3 = sway-referenced vision, firm support; condition 4 = eyes open, sway-referenced support; condition 5 = eyes closed, sway-referenced support; condition 6 = sway-referenced vision, sway-referenced support. Three trials were administered for each condition, lasting 20 s. The amplitude of the anterior-posterior CoM sway was measured in the six different conditions [57]. Each trial was scored from 0 to 100, with higher scores indicating better balance (i.e., reduced CoM oscillations). A score of 0 was assigned to a trial marked as a “fall” (due to a stepping reaction, to the participant’s hands touching the surroundings or to the participant falling and being supported by the safety jacket during the trial). In contrast, a score of 100 was assigned to complete stability (i.e., no COM sway, never achievable in practice). A mean score is calculated for each of the six SOT conditions, and a 0–100 composite score (SOT composite) is assigned to the overall test (the higher the SOT composite, the lower the COM sway).

### 2.5. Data Analysis and Statistics

In the instrumental assessment of cervical proprioception, accuracy and precision indexes were calculated for each tested direction [58].

Accuracy was expressed by the constant error [58], the within-subjects mean JPE (i.e., the mean value of the JPE from the four trials collected for each direction). Precision was expressed as the variable error [58], calculated as the within-subjects standard deviation of the JPE for each direction of movement. Note that whereas variable error is always positive, constant error can be either positive or negative depending on the participant.

Accuracy and precision indexes were calculated for the three JPE*int-components* and the associated unintended components (i.e., JPE*frontal* and JPE*sagittal* or JPE*horizontal*).

Mean sample accuracy and precision indices for the patients and control group were computed based on the participants’ constant and variable errors.

Demographic and clinical data were summarized using median and range. Fisher’s exact test was used to compare gender distribution in patients and controls. Wilcoxon’s rank sum test with continuity correction was used to compare age in patients and controls.

Linear mixed-effects models [59] were used to test whether the various JPEs (response variable) differed between groups (i.e., controls vs. patients) and the tested directions. The regression model also included the interaction between the group and movement direction. The analysis assessed if the sample JPE differed between DM1 patients and controls and across directions (i.e., extension, flexion, left rotation, and right rotation).

The linear models’ assumptions of normally distributed and homogeneous residuals were checked graphically with the quantile-quantile plot and residual-predicted plot, respectively [59]. In case these assumptions were violated, response variables were transformed. In particular, data on JPE*int-component* precision and JPE*frontal* precision were ln-transformed.

To determine the statistical significance of the fixed effects (i.e., group, direction and their interaction), a Type III analysis of variance (ANOVA) using Satterthwaite’s method was computed on the linear mixed-effects models. Least-squares means were calculated for post hoc testing and graphical purposes. Satterthwaite’s approach has also been used for post hoc testing [60].

The significance level was set at 0.05. The Holm correction for multiplicity was applied to the post hoc tests.

The Spearman correlation coefficient was used to detect any association between accuracy and precision indexes of head repositioning, clinical measures, the number of falls, and the measures from the instrumental balance assessment.

Statistical analyses were run in R version 4.3.1.

## 3. Results

### 3.1. Participants

Clinical details on the 16 DM1 patients recruited and results from the balance instrumental assessment are reported in Table 1. As anticipated, the sample of DM1 patients recruited here is the same as analyzed in a previous article from our group [20]. No differences were found for gender distribution (Fisher’s exact test: *p* = 0.722) and age (Wilcoxon’s rank sum test with continuity correction: *p* = 0.096) between patients (females/males: 10/6; median age 41.5 years, range 21–47 years) and controls (8/8; 34.5 years, 30–49 years).

### 3.2. Balance Instrumental Assessment

Table 1 reports the results from the balance instrumental assessment (i.e., SOT composite and scores from conditions 1 to 6) of the sample of 16 DM1 patients.

### 3.3. Cervical Proprioception Instrumental Assessment

#### 3.3.1. JPE*int-component* Accuracy

**Figure 1:**
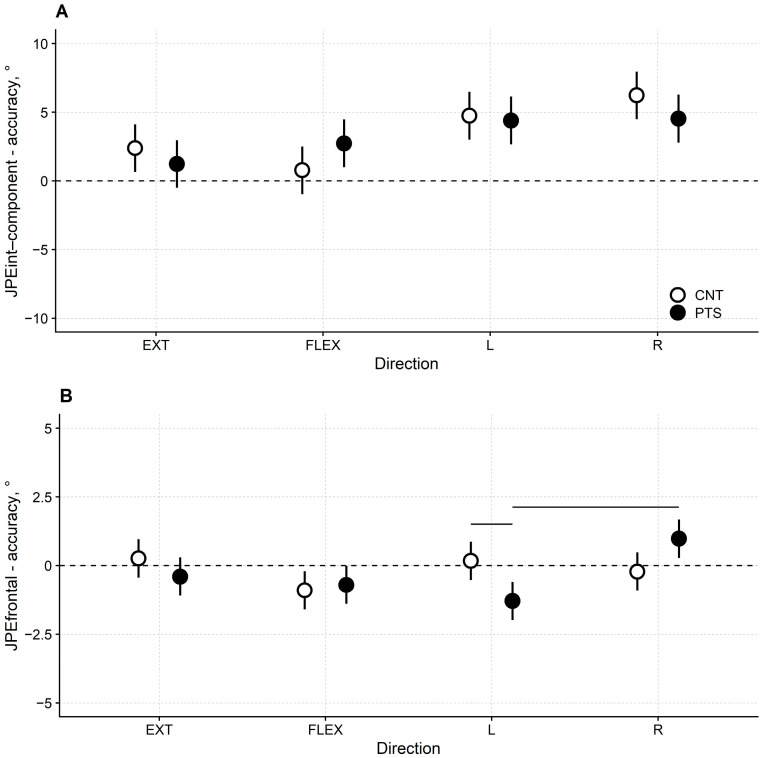
(**A**) Least-squares means and their 95% CI for DM1 patients (black dots) and healthy controls (white dots) of the JPE*int-component* accuracy and (**B**) the JPE*frontal* accuracy in the extension (EXT), flexion (FLEX), left rotation (L) and right rotation (R) trials. Horizontal bars mark a significant difference at *p* < 0.05 between paired comparisons.

The accuracy of the JPE*int-component* was comparable in DM1 patients and controls for all the intended directions of neck movement (i.e., flexion, extension, right rotation, and left rotation; Figure 1A and Table 2), indicating that the primary repositioning error was similar in the two groups.

On average, both groups slightly overshot the target position in any of the four directions, more markedly when asked to rotate the head in the left–right direction.

ANOVA resulted in a significant “direction” factor (F(3, 90) = 14.7, *p* < 0.001), while the “group” factor (F(1, 30) = 0.1, *p* = 0.733) and the “direction × group” interaction (F(3, 90) = 2.7, *p* = 0.05) were not.

In patients and controls, the JPE*int-component* was significantly larger for left and right rotations than for flexion and extension. For extension, the mean and 95%CI for the JPE*int-component* were: 1.24° [−0.501°, 2.97°] and 2.39° [0.65°, 4.13°] in DM1 patients and healthy controls, respectively; for flexion 2.74° [1.00°, 4.47°] and 0.78° [−0.960°, 2.52°], respectively; for left rotation 4.40° [2.66°, 6.14°] and 4.75° [3.01°, 6.49°], respectively; for right rotation 4.54° [2.80°, 6.28°] and 6.23° [4.49°, 7.97°], respectively. See Table S1 in Supplementary Materials, Section S1, for details on post hoc tests.

#### 3.3.2. JPE*frontal* Accuracy

The accuracy of the JPE*frontal* (Figure 1B and Table 2) was similar between patients and controls in flexion and extension trials. However, the JPE*frontal* was larger in patients when the head was rotated to the left or the right.

When intentionally performing a left rotation on the horizontal plane, the patients also unintentionally performed a side-bending to the left in the frontal plane (the mean and 95%CI for the JPE*frontal* were −1.29° [−1.99°, −0.60°]). On the contrary, on average, this error in the frontal plane did not differ statistically from 0° in controls (0.17° [−0.53°, 0.87°]).

When repositioning to the right, the patients showed an unintended side-bending to the right in the frontal plane (0.98° [0.28°, 1.67°]). Again, the controls’ JPE*frontal* was negligible and, on average, not different from 0° (−0.22° [−0.91°, 0.48°]).

According to ANOVA, the “direction” factor (F(3, 120) = 4.47, *p* = 0.005) and the “direction x group” interaction (F(3, 120) = 5.25, *p* = 0.002) were significant, while the “group” factor (F(1, 120) = 0.54, *p* = 0.464) was not.

Results from post hoc tests are reported in Table S1 in the Supplementary Materials, Section S1.

Mean JPE*frontal* accuracy differed between left and right rotations in patients (*p* < 0.001) but not in controls. In addition, when rotating the head in the horizontal plane, JPE*frontal* was significantly larger in patients than in controls when turning to the left, only (*p* = 0.028).

#### 3.3.3. JPE*int*-*component* Precision

**Figure 2:**
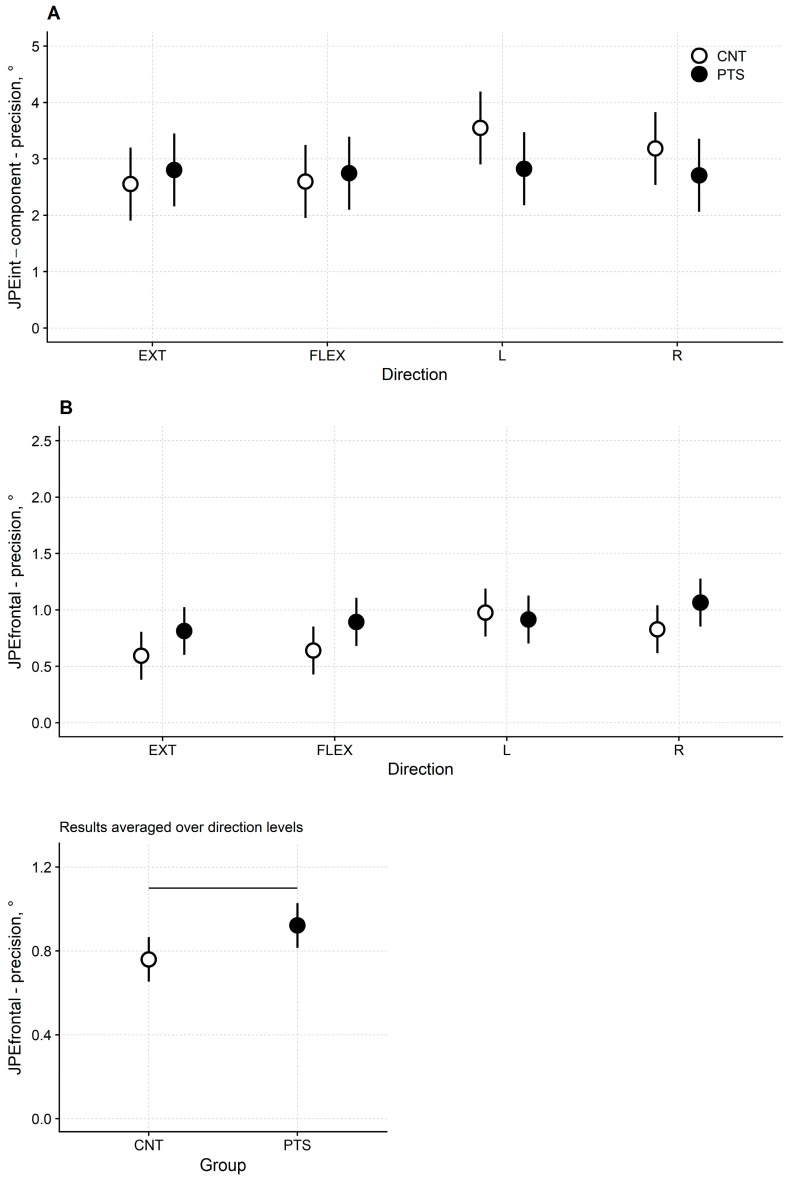
(**A**) Least-square means and 95% CI for DM1 patients (black dots) and healthy controls (white dots) of the JPE*int-component* precision and (**B**) the JPE*frontal* precision in the extension (EXT), flexion (FLEX), left rotation (L) and right rotation (R) trials. Lower panel: JPE*frontal* precision for DM1 patients and healthy controls averaged over directions. The horizontal bar marks a significant difference at *p* < 0.05. Significance testing for JPE*int-component* and JPE*frontal* precision has been run on ln-transformed data to comply with the non-normality and heteroscedasticity of the residuals. However, in the plot, non-transformed data are represented for the JPE*int-component* and JPE*frontal* to allow comparisons.

JPE*int-component* precision was comparable across the four movements and the two groups of participants (Figure 2A and Table 3). Significance testing showed that neither the “direction” factor (F(3, 90) = 1.28, *p* = 0.288) nor the “group” factor (F(1, 30) = 0.74, *p* = 0.396) nor the “direction × group” interaction (F(3, 90) = 1.37, *p* = 0.258) was significant.

#### 3.3.4. JPE*frontal* Precision

The variable error of JPE*frontal* was slightly higher (i.e., precision was lower) in patients than in controls for flexion-extension and for the right rotation trials (Figure 2B upper panel and Table 3). For extension, the mean and 95%CI for the JPE*frontal* were 0.81° [0.60°, 1.03°] and 0.59° [0.38°, 0.81°] in DM1 patients and healthy controls, respectively. For flexion 0.89° [0.68°, 1.11°] and 0.64° [0.43°, 0.85°], respectively. For left rotation 0.92° [0.70°, 1.13°] and 0.98° [0.76°, 1.19°], respectively. For right rotation 1.07° [0.85°, 1.28°] and 0.83° [0.62°, 1.04°], respectively.

ANOVA confirmed this finding. Factors “direction” (F(3, 120) = 3.74, *p* = 0.013) and “group” (F(1, 120) = 4.31, *p* = 0.040) were significant, while the “direction × group” interaction (F(3, 120) = 0.97, *p* = 0.409) was not.

Regarding the between-groups difference, ANOVA showed that, irrespectively of the movement direction, the overall precision of JPE*frontal* was lower for DM1 patients than for controls (patient’s mean [95%CI] variable error: 0.92° [0.82°, 1.03°]; controls: 0.76° [0.65°, 0.87°]; Figure 2B lower panel).

Results from post hoc tests are reported in Table S2 in the Supplementary Materials, Section S1.

#### 3.3.5. JPE Accuracy and Precision on the Sagittal and Horizontal Planes

Accuracy and precision of the unintended components of JPE on the sagittal and horizontal planes have also been assessed as a complementary analysis. No difference between patients and controls was apparent.

Results of the analysis of JPE*sagittal* and JPE*horizontal* are reported in Table 2 and Table 3 and described in detail in the Supplementary Materials, Section S2.

### 3.4. Association between JPEs, Clinical Measures and Instrumental Balance Measures

The previous analysis has shown that the accuracy and precision of JPE*frontal* differed between patients and controls, with DM1 patients showing less accuracy and less precision in the frontal plane than healthy controls in the repositioning task. Therefore, the Spearman correlation coefficient was used to detect any association between the amount of JPE*frontal* constant and variable errors, clinical measures (i.e., MIRS and DHIsf), the number of falls in the preceding 12 months, and the measures from the instrumental balance assessment (i.e., the SOT composite score and the average scores from each of the six SOT conditions). Table 4 reports the strength of the associations (Spearman ρ and *p*-value). No significant correlations were found.

Results of the analysis of JPE*3D* are provided in the Supplementary Materials, Section S3.

## 4. Discussion

In this observational cross-sectional study, we compared cervical proprioception in 16 DM1 patients and a sample of age- and gender-matched controls. Cervical proprioception was assessed through an HTT repositioning test. In the DM1 patients, clinical and instrumented balance measures were also collected. The ultimate aim of this study was to investigate if an impairment in the somatosensory afferents from the neck muscles, indicated by an impairment of neck proprioception, is associated with standing balance impairment in DM1 patients.

The HTT revealed lower accuracy (i.e., a higher constant error) and lower precision (i.e., a larger variable error) in DM1 patients compared to healthy controls. In particular:

- The accuracy on the frontal plane (i.e., JPE*frontal*) was decreased in DM1 patients: left and right rotations were associated with an unintended side-bending towards the side of rotation that was significantly greater than 0°.
- When patients and controls were compared, the ANOVA modelling gave significance (lower patients’ accuracy) of JPE*frontal* only for the left rotation.
- The overall precision of repositioning in the frontal plane, whichever side of head rotation, was lower in the DM1 patients than in the controls.

In the samples of DM1 patients and controls recruited here, the HTT repositioning test errors ranged, on average, from 1° to 6° for the JPE*int-component*. In contrast, the unintended component errors were roughly 1° or less. Although other studies have used different measuring devices, the overall amplitude of repositioning errors in our study was similar to those from previous HTT repositioning tests involving healthy individuals [61,62] and/or participants with neck disorders [61,63] (e.g., non-traumatic neck pain, cervicogenic headache or whiplash). In the literature, head repositioning tests for measuring cervical proprioception were primarily used in patients with pain syndromes involving the neck (e.g., chronic neck pain [64,65] and whiplash [66,67]) and less frequently in patients with cervicogenic dizziness [68] or vestibular impairments [66]. No previous study investigated cervical proprioception in neuromuscular disorders. Also, prior research did not consider the individual components of JPE projected in the horizontal, frontal, and sagittal planes.

Both DM1 patients and healthy controls showed higher errors of the JPE*int-component* accuracy in the trials performed on the horizontal plane (i.e., left and right rotations) than in those performed on the sagittal plane (i.e., flexion and extension). The evidence that participants most commonly overshoot the target in HTT repositioning tests has already been pointed out in previous studies [69,70]. This result and the fact that overshooting seems to be a general phenomenon in joint repositioning testing [71,72,73] were discussed in detail in our previous paper [53]. Here, it is noteworthy that the DM1 patients did not differ from the controls when overshooting was concerned.

As reported above, HTT repositioning tests the neck’s proprioception. However, to correctly conclude that poor performance on an HTT repositioning test flags a proprioceptive impairment, it must be shown that the performance on the test is not secondary to other impairments, such as articular limitations or muscle weakness.

Compared to controls, the greater side-bending found in the DM1 patients could be due to mechanical changes in the cervical joints caused by alterations of their bony and cartilage components. However, this hypothesis seems unlikely here since none of the DM1 had a history of chronic neck pain. The operator-assisted head motions during HTT testing were entirely painless. Also, no participant showed any limitation of cervical joint motion in the range considered in this test. Last, any existing limitation of motion due to osteoarticular reasons would have likely affected both the autonomous and the operator-assisted head motions (the latter representing the repositioning target). However, despite these considerations, some local osteoarticular impairments could not be ruled out.

Unintended side-bending occurred during left and right rotations of the head. This could be due to the weakness of neck muscles, resulting in the poor support of the rotated head against gravity. However, if neck weakness plays a role, DM1 patients should have also shown greater target overshooting in flexion (head drop braked by neck extensors) and undershooting in extension (facing the head weight) and perhaps in axial rotations (facing head inertia) compared to healthy controls.

A vestibular cause could also be hypothesized. Previous studies have described auditory and vestibular abnormalities in DM1 patients, suggesting extensive labyrinthine deficits [18], and widespread grey matter and white matter alterations [74,75]. However, it is unlikely that such a widespread vestibular involvement could produce an uniplanar and unidirectional head repositioning error, i.e., the higher JPE*frontal* for left–right rotations. On the contrary, there is no evidence of any focal vestibular impairment in DM1 (e.g., afferents from the lateral and/or posterior semicircular canals [76]).

Therefore, the results seem consistent with the original hypothesis of the present study, e.g., one of the neck proprioception impairments possibly resulting from the alteration of the intrafusal muscle fibers in DM1. However, given that all the aforementioned hypotheses are plausible, further physiologic and pathoanatomical knowledge is still needed to allow firm conclusions.

As anticipated, the earliest descriptions of histological abnormalities in the muscle spindles of myotonic dystrophy patients date back to the 1960s [39]. In particular, studies performed in the 1970s have described the longitudinal fragmentation of intrafusal fibers due to degenerative and regenerative changes [40], involving both the polar and the equatorial regions [41] and affecting, in particular, nuclear bag fibers [77]. Also, alterations in the spindles’ innervation have been reported [78], such as the proliferation and structural abnormality (e.g., incomplete contacts between the nerve terminals and the muscle fiber fragments, abnormal shapes of the terminals) of both sensory and motor nerve terminals [42]. Finally, it has been reported, through an electrophysiological study performed in vitro, the impairment of afferent units in muscle spindles from patients with myotonic dystrophy [42]. However, the present study is the first in vivo evaluation of joint proprioception in DM1 patients.

When the head is turned left and right, the exact mechanism causing higher constant and variable errors on the frontal plane remains unknown. Furthermore, humans have no “pure” lateral neck flexors, as all neck muscles combine actions on the frontal, sagittal, and horizontal planes [33,79]. Muscles with the most direct side-bending action are the Sternocleidomastoideus, Scalenus anterior, and Obliquus capitis superior [80]. Also, due to the morphology of facet and uncovertebral joints, side-bending is always coupled with axial rotation and this coupling pattern changes along the cervical spine [81,82,83]. In this regard, not all neck muscles seem to be affected similarly in DM1. A recent MRI study on a cohort of 134 DM1 patients revealed that the Sternocleidomastoideus is mostly atrophic. Conversely, the Trapezius and the Paraspinalis are commonly replaced by fat [84]. Instead, the involvement of the Obliquus capitis inferior and the Scalenes seems less frequent [85]. It can be speculated that such a dispersed, non-uniform distribution of muscle alterations may result in movement errors occurring more frequently in certain planes.

Another element that needs further clarification is the absence of a correlation between the magnitude of the constant and the variable errors in DM1 patients and their static balance impairment. Patients underperformed remarkably in balance testing, as described in our previous article [20].

Neck proprioception plays a role in standing balance regulation [86]. An impairment of the neck’s muscles might affect the vestibular system’s function, given the connection between head stabilization and vestibular information [44]. As for the absence of correlation between balance and neck proprioception revealed by our results, it should be noted that, in the first place, only standing balance in the sagittal plane, albeit measured with a valid procedure, was taken into consideration here (i.e., the maximum amplitude of oscillation of the COM during the EquiTest SOT). Additional posturographic indices, like the path of the center of pressure or the COM’s speed, might be considered in further studies. Furthermore, mediolateral oscillations may also help assess stability [87,88,89]. Moreover, no head motions are requested during typical posturography, although sensitization through head-shaking motions has been proposed [90]. It is worth mentioning that, to the authors’ knowledge, only one study has tried to correlate results from cervical proprioception and posturography, showing weak-to-moderate correlations between the balance scores and the JPE in the right and left rotations in patients with whiplash-associated disorders [67].

Static balance testing is inherently limited. Dynamic balance (i.e., the ability to reach the upright stance or to move in an upright stance without falling or to maintain balance during walking) should also be considered because it involves specific forms of neural control [91]. In this regard, it is worth mentioning that balance impairments may result in falls, mostly during walking [89].

Future research on balance in DM1 patients might follow two main lines of investigation: one biomechanical (like the one depicted here) and the other pathoanatomical.

Regarding the former (i.e., biomechanical investigations), the role of cervical proprioception in DM1 patients might be tested in more challenging movements, for instance, broader or faster head rotations, compared to those tested here. Methods might include movements following more complex trajectories or cyclic repeats (like in head-shaking paradigms). Impairments of cervical position sense might emerge when a high interaction is requested between cervical position sense, vision, vestibular senses and complex bodily motions, like in walking or when the cognitive load is increased in motor-cognitive dual-task paradigms [92].

As concerns anatomical investigations, it is worth mentioning that the anatomical studies dealing with the characterization of muscle spindles in DM1 were performed more than thirty years ago, and none analyzed the deep neck muscles: biopsies were taken from Pronator teres and Lumbricals [40], Tibialis anterior [41], Vastus lateralis and Vastus medialis [93], Extensor indices [42,77], and several muscles of the head, trunk and limbs [39,78] The affection of neck flexors is a typical (though not exclusive) feature of DM1 [94]. Therefore, future studies should better clarify the ultrastructural and functional alterations affecting muscle spindles in the deep neck muscles. Imaging studies of these muscles are also advisable, considering that, in recent years, some authors have proposed the adoption of muscle imaging biomarkers in DM1 [95]. For instance, ultrasound-based parameters of the Diaphragm have been related to the respiratory function [96] and MRI alterations of the Masseter muscles have already been proposed as indexes of aspiration risk [97]. Likewise, MRI studies targeting the involvement of neck muscles in DM1 patients might further support their role in balance impairment.

This research is not the first one discussing spindle pathology in neuromuscular diseases. Other researchers have hypothesized that proprioception can be impaired due to spindle involvement in hereditary disorders affecting muscle tissue [98]. Indeed, alterations in the structure of muscle spindles have also been described in other types of muscular dystrophies. However, results were contradictory and differed among disorders [30]. Animal models have also been developed, showing that mutant mice deprived of muscle spindles cannot support their weight and show abnormal postures [99].

However, as already recalled, no previous study tested joint proprioception in patients with DM1 or affected by other neuromuscular diseases. In this regard, in vivo investigation is not of little importance. Indeed, given the complexity of the neurophysiological processes encompassing proprioception, histological examinations only can hardly provide satisfactory results. Some authors have employed vibratory stimulation to test the functionality of muscle spindles in patients with muscular dystrophies. Their results suggested that the proprioceptive function of muscle spindles in these patients can be comparable to that of healthy individuals [100]. However, their research involved a rather heterogeneous sample of patients (three patients with Becker dystrophy, one with Duchenne dystrophy, five with myotonic dystrophy, five with facioscapulohumeral dystrophy, and six with limb–girdle dystrophy), and studies involving humans and animal models with muscular dystrophies showed that the degrees of impairment of muscle spindle may vary considerably across different diagnoses [30].

As a final note, this study gives clinicians innovative cues for DM1 patients’ rehabilitation. Indeed, the research was prompted by a previous case report suggesting that strengthening neck muscles can improve standing balance in DM1 patients [101].

The current study has some limitations worth discussing. Participants in this HTT test were instructed to move their heads, hold the position, and return to the starting position. Repositioning errors during the “hold the position” phase of the HTT test were selected as indicators of cervical proprioception [29]. Although this method of measuring cervical proprioception is the most widely used in the literature [29] other movement parameters might reflect proprioception as well. For instance, impairments might be revealed by alterations in 3D paths and trajectories (including velocity, acceleration, and jerk [53]). In particular, jerk is a promising index of motion smoothness and ataxia.

Another limitation of this study that is worth mentioning is the small sample size of only 16 individuals diagnosed with DM1. In this regard, it must be noted that DM1 is a rare disorder and that this study’s inclusion criteria were quite demanding. In particular, patients had to keep upright without assistive devices, their visual acuity had to be preserved (in a condition in which cataracts are common [102]), and patients with any previous major orthopedic surgery were excluded. Also, it is worth mentioning that previous studies investigating cervical proprioception in other populations have recruited samples of similar size [55,103,104].

As per the inclusion criteria, DM1 participants were relatively young (i.e., 21 to 47 years). On the one hand, this could be seen as a further limitation. On the other hand, this restricted age range minimized the recruitment of patients who could be affected by cervical osteoarthritis and other medical conditions possibly involving a balance impairment. Reports have described the occurrence of spine problems in DM1 [105], and previous research on individuals with cervical spondylosis [106] or with traumatic and non-traumatic neck pain [107,108] has revealed that they may show an increased JPE in repositioning tests. Moreover, regarding the primary hypothesis tested here, data from healthy humans suggest that, in the cervical muscles, spindle distribution, morphology, and density do not change with age [109].

## Figures and Tables

**Table 1:** Patients’ characteristics.

ID	Age (y)	Gender	Height (m)	Duration (y)	E Class	MIRS	DHIsf	RMI	N of falls	SOT	COND1	COND2	COND3	COND4	COND5	COND6
1	41	F	1.70	15	2	3	13	15	0	68	95	89	94	67	22	70
2	39	F	1.68	26	1	3	11	15	2	71	92	86	85	85	42	61
3	25	F	1.62	11	2	3	13	15	3	82	95	93	90	84	68	76
4	42	M	1.78	11	1	3	10	15	0	74	95	91	92	87	26	77
5	40	M	1.82	21	1	1	13	15	0	33	95	92	90	0	0	0
6	26	M	1.69	25	2	3	13	15	0	65	94	93	88	85	30	40
7	21	F	1.70	19	3	2	12	15	3	53	94	87	78	54	53	0
8	47	M	1.65	42	1	4	9	15	1	74	91	93	88	77	70	48
9	43	F	1.56	28	2	3	11	10	5	70	88	90	87	74	51	57
10	42	M	1.78	23	2	3	10	12	0	44	94	84	89	59	0	0
11	47	F	1.65	14	2	4	13	14	0	51	95	94	93	82	0	0
12	47	F	1.60	21	2	4	13	14	0	32	93	89	91	0	0	0
13	46	F	1.60	17	2	3	8	12	1	43	93	93	91	47	0	0
14	41	F	1.64	31	3	3	9	14	1	25	90	84	47	13	0	0
15	38	F	1.70	26	2	4	13	14	2	40	96	95	95	27	0	0
16	47	M	1.74	19	2	3	11	14	0	50	94	91	91	81	0	0
	41.5	F/M: 10/6	1.69	21	2	3	11.5	14.5	0.5	52.0	94	91	90	71	11	0
	(21–47)		(1.56–1.82)	(11–42)	(1–3)	(1–4)	(8–13)	(10–15)	(0–5)	(25–82)	(88–96)	(84–95)	(47–95)	(0–87)	(0–70)	(0–77)

Clinical and anthropometric characteristics of the 16 DM1 patients recruited in this study. The last row reports the patients’ sample’s median values (and range, in brackets). The ratio between the number of females and the number of males (F/M) is given in the bottom row of the third column. ID: patient’s identification number. F: female; M: male; duration: disease duration, in years; E class: class attributed according to the number of repetitions (i.e., expansion) of CTG triplets; MIRS: Muscular Impairment Rating Scale; DHIsf: Dizziness Handicap Inventory—short form; RMI: Rivermead Mobility Index; N of falls: number of falls in the 12 months before the assessment; SOT: the cumulative 0–100 composite score assigned to the overall SOT (with higher scores reflecting better performance); COND 1 to 6 refer to the six balance conditions tested during SOT (mean score across three repetitions, rounded to the nearest integer); COND 1 = eyes open, fixed support; COND 2 = eyes closed, fixed support; COND 3 = sway-referenced vision, fixed support; COND 4 = eyes open, sway-referenced support; COND 5 = eyes closed, sway-referenced support; COND 6 = sway-referenced vision, sway-referenced support.

**Table 2:** Accuracy of cervical repositioning for DM1 patients and healthy controls.

		JPE*int-component* Accuracy		JPE*frontal* Accuracy		JPE*sagittal* Accuracy		JPE*horizontal* Accuracy	
Group	Direction	Mean	95% CI	Mean	95% CI	Mean	95% CI	Mean	95% CI
CNT	Extension	2.39	[0.65, 4.13]	0.26	[−0.437, 0.96]	2.39	[0.65, 4.13]	0.53	[−0.22, 1.27]
DM1	Extension	1.24	[−0.50, 2.97]	−0.40	[−1.10, 0.30]	1.24	[−0.50, 2.97]	0.78	[0.03, 1.52]
CNT	Flexion	0.78	[−0.96, 2.52]	−0.90	[−1.60, −0.21]	0.78	[−0.96, 2.52]	−0.80	[−1.55, −0.05]
DM1	Flexion	2.74	[1.00, 4.47]	−0.70	[−1.40, −0.01]	2.74	[1.00, 4.47]	−0.51	[−1.26, 0.23]
CNT	Left rotation	4.75	[3.01, 6.49]	0.17	[−0.53, 0.87]	0.62	[−0.65, 1.90]	4.75	[3.01, 6.49]
DM1	Left rotation	4.40	[2.66, 6.14]	−1.29	[−1.99, −0.60]	0.82	[−0.45, 2.10]	4.40	[2.66, 6.14]
CNT	Right rotation	6.23	[4.49, 7.97]	−0.22	[−0.91, 0.48]	−1.52	[−2.28, −0.24]	6.23	[4.49, 7.97]
DM1	Right rotation	4.54	[2.80, 6.28]	0.98	[0.28, 1.67]	−0.80	[−2.07, 0.48]	4.54	[2.80, 6.28]

Results on the accuracy of the HTT repositioning test for DM1 patients and healthy controls with respect to the four tested directions (i.e., extension, flexion, left rotation, and right rotation). JPE*int-component*: the component of JPE belonging to the ideal plane of the movement’s direction. JPE*frontal*: the component of JPE belonging to the frontal plane. JPE*sagittal*: the component of JPE belonging to the sagittal plane. JPE*horizontal*: the component of JPE belonging to the horizontal plane. CNT: healthy controls. DM1: DM1 patients. Mean: the group average of the participant’s mean JPE accuracy (as calculated for JPE*int-component*, JPE*frontal*, JPE*sagittal*, and JPE*horizontal*)—95% CI: 95% Confidence Intervals. In this table, for movements performed in the sagittal plane (i.e., extension and flexion trials), values for JPE*sagittal* are repeated in the JPE*int-component* column because, in these trials, the JPE*sagittal* corresponds to the JPE*int-component*. Likewise, for movements performed in the horizontal plane (i.e., left and right rotation trials), values for JPE*horizontal* are repeated in the JPE*int-component* column because, in these trials, the JPE*horizontal* corresponds to the JPE*int-component*.

**Table 3:** Precision of cervical repositioning for DM1 patients and healthy controls.

		JPE*int-component* Precision		JPE*frontal* Precision		JPE*sagittal* Precision		JPE*horizontal* Precision	
Group	Direction	Mean	95% CI	Mean	95% CI	Mean	95% CI	Mean	95% CI
CNT	Extension	2.55	[1.91, 3.20]	0.593	[0.38, 0.81]	2.55	[1.91, 3.20]	1.07	[0.80, 1.33]
DM1	Extension	2.80	[2.15, 3.45]	0.813	[0.60, 1.03]	2.80	[2.15, 3.45]	1.10	[0.83, 1.36]
CNT	Flexion	2.60	[1.95, 3.25]	0.640	[0.43, 0.85]	2.60	[1.95, 3.25]	1.02	[0.76, 1.28]
DM1	Flexion	2.75	[2.10, 3.39]	0.893	[0.68, 1.11]	2.75	[2.10, 3.39]	1.20	[0.94, 1.47]
CNT	Left rotation	3.55	[2.90, 4.20]	0.98	[0.76, 1.19]	1.78	[1.37, 2.19]	3.55	[2.90, 4.20]
DM1	Left rotation	2.82	[2.18, 3.47]	0.92	[0.70, 1.13]	1.44	[1.03, 1.85]	2.82	[2.18, 3.47]
CNT	Right rotation	3.18	[2.54, 3.83]	0.83	[0.62, 1.04]	1.44	[1.03, 1.84]	3.18	[2.54, 3.83]
DM1	Right rotation	2.71	[2.06, 3.36]	1.07	[0.85, 1.28]	1.78	[1.37, 2.19]	2.71	[2.06, 3.36]

Results on the precision of the HTT repositioning test for DM1 patients and healthy controls with respect to the four tested directions (i.e., extension, flexion, left rotation, and right rotation). JPE*int-component*: the component of JPE belonging to the same plane of the movement’s direction. JPE*frontal*: the component of JPE belonging to the frontal plane. JPE*sagittal*: the component of JPE belonging to the sagittal plane. JPE*horizontal*: the component of JPE belonging to the horizontal plane. CNT: healthy controls. DM1: DM1 patients. Mean the group average of the participant’s mean JPE precision (as calculated for JPE*int-component*, JPE*frontal*, JPE*sagittal*, and JPE*horizontal*)—95% CI: 95% Confidence Intervals. In this table, for movements performed in the sagittal plane (i.e., extension and flexion trials), values for JPE*sagittal* are repeated in the JPE*int-component* column because, in these trials, the JPE*sagittal* corresponds to the JPE*int-component*. Likewise, for movements performed in the horizontal plane (i.e., left and right rotation trials), values for JPE*horizontal* are repeated in the JPE*int-component* column because, in these trials, the JPE*horizontal* corresponds to the JPE*int-component*. Data on JPE*int-component* and JPE*frontal* precision were ln-transformed to comply with the normality and homoscedasticity of the residuals. However, the results in Table 3 for the JPE*int-component* and JPE*frontal* are given on the response scale (i.e., not as ln-transformed) to allow comparisons.

**Table 4:** Correlation between frontal joint position error, clinical measures and instrumental balance measures.

	JPE*frontal* Accuracy		JPE*frontal* Precision	
	Spearman ρ	*p*-Value	Spearman ρ	*p*-Value
MIRS	0.26	0.322	0.33	0.205
DHIsf	−0.22	0.415	0.14	0.616
N. of falls	−0.17	0.540	0.20	0.460
SOT	−0.28	0.297	−0.19	0.474
COND 1	0.15	0.578	−0.41	0.117
COND 2	−0.20	0.463	0.00	0.996
COND 3	−0.07	0.790	−0.25	0.351
COND 4	0.01	0.957	−0.11	0.696
COND 5	−0.25	0.351	−0.16	0.561
COND 6	−0.15	0.582	−0.29	0.284

Correlation between the accuracy and precision of JPE*frontal* in DM1 patients, clinical measures, number of falls in the preceding 12 months, and measures from the instrumental balance assessment. JPE*frontal*: the component of JPE belonging to the frontal plane. MIRS: Muscular Impairment Rating Scale. DHIsf: Dizziness Handicap Inventory—short form. N of falls: number of falls in the 12 months before the assessment. SOT composite: the cumulative 0–100 composite score assigned to the overall SOT. SOT conditions 1 to 6 refer to the six balance conditions administered to each participant during the SOT. SOT condition 1 = eyes open, firm support. SOT condition 2 = eyes closed, firm support. SOT condition 3 = sway-referenced vision, firm support. SOT condition 4 = eyes open, sway-referenced support. SOT condition 5 = eyes closed, sway-referenced support. SOT condition 6 = sway-referenced vision, sway-referenced support.

## Data Availability

The data supporting this study’s findings are available from the authors upon reasonable request.

## Supplementary Materials

The following supporting information can be downloaded at: https://www.mdpi.com/article/10.3390/jcm13164685/s1, Section S1: Table S1: Post-hoc analysis of head repositioning accuracy in the head-to-target (HTT) test in DM1 patients and controls, Table S2: Post-hoc analysis of head repositioning precision in the head-to-target (HTT) test in DM1 patients and controls; Section S2: Figure S1: (A) Least-squares means and their 95% CI for DM1 patients (black dots) and healthy controls (white dots) of the JPE*sagittal* accuracy and (B) the JPE*horizontal* accuracy in the extension (EXT), flexion (FLEX), left rotation (L) and right rotation (R) trials, Figure S2: (A) Least-squares means and their 95% CI for DM1 patients (black dots) and healthy controls (white dots) of the JPE*sagittal* precision and (B) the JPE*horizontal* precision in the extension (EXT), flexion (FLEX), left rotation (L) and right rotation (R) trials; Section S3: Table S3: JPE3D precision
