# Supplementary material for: In Myotonic Dystrophy Type 1 Head Repositioning Errors Suggest Impaired Cervical Proprioception
Source: J Clin Med. 2024 Aug 9;13(16):4685. doi: 10.3390/jcm13164685 (PMC11355930; doi:10.3390/jcm13164685)
Supplement: Supplementary file 1 [file jcm-13-04685-s001.zip › jcm-3088974-supplementary.pdf]

In Myotonic Dystrophy Type 1 head repositioning errors suggest impaired cervical proprioception

Stefano Scarano [1,2], Antonio Caronni [1,2,*], Elena Carraro [3], Carola Rita Ferrari Aggradi [3], Viviana Rota [2], Chiara Malloggi [2], Luigi Tesio [2] and Valeria Ada Sansone [1,3]

[1] Department of Biomedical Sciences for Health, University of Milan, 20133 Milan, Italy; s.scarano@auxologico.it (S.S.); valeria.sansone@centrocliniconemo.it (V.A.S.)
[2] IRCCS Istituto Auxologico Italiano, Department of Neurorehabilitation Sciences, Ospedale San Luca, 20122 Milan, Italy; v.rota@auxologico.it (V.R.); chiaradiletta.malloggi@ospedaleniguarda.it (C.M.); l.tesio@auxologico.it (L.T.)
[3] The NeMO Clinical Center in Milan, Neurorehabilitation Unit, University of Milan, 20162 Milan, Italy; elena.carraro@centrocliniconemo.it (E.C.); carola.ferrari@unimi.it (C.R.F.A.)
* Correspondence: a.caronni@auxologico.it

Supplementary materials, Section S1

Supplementary Table S1. Post-hoc analysis of head repositioning accuracy in the head-to-target (HTT) test in DM1 patients and controls.

	JPE*int-component* constant error	JPE*frontal* constant error
Predictor: direction		
Contrasts	p-value	p-value
Extension vs Flexion	0.935	0.158
Extension vs Left rotation	<0.001*	0.498
Extension vs Right rotation	<0.001*	0.498
Flexion vs Left rotation	0.001*	0.498
Flexion vs Right rotation	<0.001*	0.006*
Left rotation vs Right rotation	0.490	0.043*
Predictor: direction x group		
Contrasts	p-value	p-value
Extension, CNT vs DM1	1.000	0.747
Flexion, CNT vs DM1	0.822	1.000
Left rotation, CNT vs DM1	1.000	0.028*
Right rotation, CNT vs DM1	0.822	0.108
CNT, Extension vs Flexion	0.822	0.108

DM1, Extension vs Flexion	0.822	1.000
CNT, Left rotation vs Right rotation	0.822	1.000
DM1, Left rotation vs Right rotation	1.000	<0.001*

Post-hoc tests are only used to evaluate the significant predictors from the ANOVA analysis (i.e., direction and the direction x group interaction; see main text). DM1: Myotonic Dystrophy type 1 patients. CNT: controls.

Supplementary Table S2. Post-hoc analysis of head repositioning precision in the head-to-target (HTT) test in DM1 patients and controls.

	JPE*frontal* variable error
Predictor: direction	
Contrast	p-value
Extension vs Flexion	0.537
Extension vs Left rotation	0.018*
Extension vs Right rotation	0.051
Flexion vs Left rotation	0.379
Flexion vs Right rotation	0.537
Left rotation vs Right rotation	0.672

Same abbreviations as in Table S1.

Supplementary materials, Section S2

Analysis of JPE accuracy and precision on the sagittal and horizontal planes

For JPE*sagittal* accuracy (Figure S1, panel A), a significant "direction" factor was found ($F(1, 30) = 32.95$, $p < 0.001$) because participants produced a positive JPE in the sagittal plane when rotating to the left (i.e., the neck was extended) and a negative JPE*sagittal* when rotating to the right (i.e., the neck was flexed). On the contrary, the "group" factor and the "direction x group" interaction were not significant.

Similarly, a significant "direction" factor was also found for JPE*horizontal* ($F(1, 60) = 12.27$, $p < 0.001$). When extending their neck, participants produced a positive JPE*horizontal* (i.e., they turned their head to the right), whereas it was negative in the flexion trials (Figure S1, panel B).

No difference due to the movement direction or group membership was found for the JPE precision on the sagittal and horizontal planes (Figure S2, panels A and B).

Supplementary Figure S1

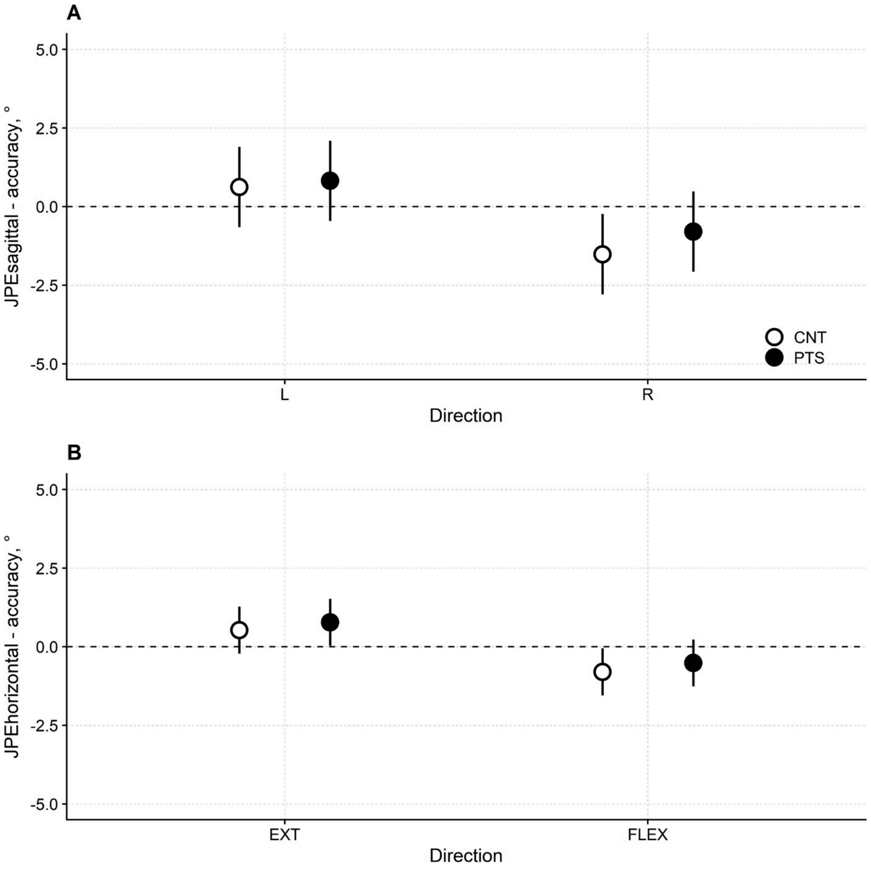

Supplementary Figure S1. **(A)** Least-squares means and their 95% CI for DM1 patients (black dots) and healthy controls (white dots) of the JPE*sagittal* accuracy and **(B)** the JPE*horizontal* accuracy in the extension (EXT), flexion (FLEX), left rotation (L) and right rotation (R) trials.

Supplementary Figure S2

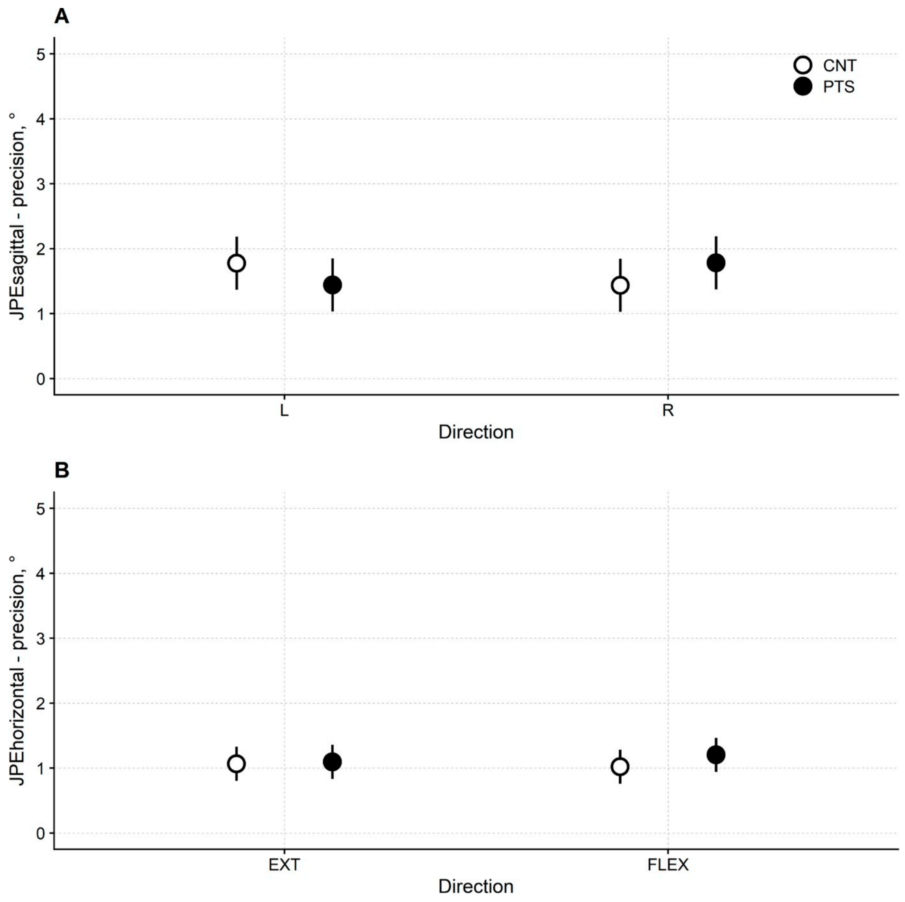

Supplementary Figure S2. **(A)** Least-squares means and their 95% CI for DM1 patients (black dots) and healthy controls (white dots) of the JPE*sagittal* precision and **(B)** the JPE*horizontal* precision inthe extension (EXT), flexion (FLEX), left rotation (L) and right rotation (R) trials.

Supplementary materials, Section S3

JPE*3D* returns the overall angular repositioning error by integrating the errors on the three planes into a single index (see main text).

For each participant, the JPE*3D* was calculated for each of the four repetitions of the four directions (as for the three planar JPEs). Contrary to the planar JPEs, JPE*3D* is always positive.

In order to improve the residual normality and homogeneity of variance, the response variable has been log-transformed for the analysis of JPE*3D* accuracy and it has been square root-transformed for the analysis of JPE*3D* precision.

JPE3D accuracy

The four JPE*3D* were averaged for each participant, and a participant's accuracy index (i.e., constant error, the within-subject mean JPE*3D*) was obtained.

The JPE*3D* constant error was comparable in DM1 patients and controls for all the intended directions of neck movement.

ANOVA resulted in a significant "direction" factor ($F(3, 90) = 8.9$, $p < 0.001$), while the "group" factor ($F(1, 30) = 0.1$, $p = 0.730$) and the "direction x group" interaction ($F(3, 90) = 1.5$, $p = 0.210$) were not.

Also, differently from the JPE*int-component*, no significant interaction between group and direction was observed.

JPE3D precision

As for the three planar JPEs, the JPE*3D* variable error was calculated as the within-subjects standard deviation of JPE*3D* for each direction of movement.

ANOVA resulted in a significant "group" factor ($F(1, 120) = 5.0$, $p = 0.027$), while the "direction" factor ($F(3, 120) = 0.6$, $p = 0.610$) and the "direction x group" interaction ($F(3, 120) = 2.1$, $p = 0.100$) were not.

Unexpectedly indeed the overall precision was higher (i.e., the JPE*3D* dispersion lower) in DM1 patients than in healthy controls: the mean and 95%CI for the overall JPE*3D* precision is 1.89° [1.62°, 2.16°] and 2.28° [2.01°, 2.55°] for DM1 patients and healthy controls, respectively.

It is useful to look at the precision indices on the three planes in order to comprehend this paradoxical finding (i.e. Figure 2 in the main manuscript and Figure S2 in the Supplementary materials).

Figure S2 in the Supplementary materials shows similar variable errors in controls and patients in the sagittal and horizontal planes when these are planes of unintended movement, i.e., the planes in which ideally no movement should have happened. In contrast to the JPE*3D* variable error, the variable error of the unintentional movement on the frontal plane is higher in patients than in controls (Figure 2B in the main text). Nevertheless, a glance at Figure 1A (the main text) reveals that when the head is moved in the horizontal plane, the variable error —that is, the JPE*int-component* in the horizontal plane— is greater in controls for both left and right rotations.

When taken into account as a whole, this greater JPE*int-component* variable error may outweigh the JPE*frontal* variable error, resulting in a bigger JPE*3D* variable error.

It is currently unknown why the JPE*int-component* variable error is higher in controls than in patients, even if it is not statistically significant. However, a higher JPE*int-component* variable error in controls could have been found if they, as may be expected, moved faster than patients, given the known inverse relationship between movement speed and precision.

<u>*Association between JPE3D, clinical measures and instrumental balance measures*</u>

The analysis has shown a difference between DM1 patients and healthy controls with respect to JPE*3D* precision. For this reason, the correlation between the JPE*3D* precision index and clinical and instrumental balance measures has been investigated. Results are reported in Table S3. No correlation has been found (as for the analysis of JPE*frontal* accuracy and precision, see main text).

Supplementary Table S3

	JPE*3D* precision	
	Spearman ρ	p-value
MIRS	0.17	0.523
DHIsf	0.39	0.132
N. of falls	0.15	0.579
SOT	0.01	0.983
COND 1	0.22	0.407
COND 2	0.29	0.274
COND 3	0.27	0.309
COND 4	-0.22	0.414
COND 5	0.18	0.511
COND 6	0.13	0.632

Supplementary Table S3. Correlation between the precision of JPE*3D* in DM1 patients, clinical measures, number of falls in the preceding 12 months, and measures from the instrumental balance assessment. JPE*3D*: the angular error in repositioning on whichever plane the error is measured on. MIRS: Muscular Impairment Rating Scale. DHIsf: Dizziness Handicap Inventory – short form. N of falls: number of falls in the 12 months before the assessment. SOT composite: the cumulative 0–100 composite score assigned to the overall SOT. SOT conditions 1 to 6 refer to the six balance conditions administered to each participant during the SOT. SOT condition 1 = eyes open, firm support. SOT condition 2 = eyes closed, firm support. SOT condition 3 = sway-referenced vision, firm support. SOT condition 4 = eyes open, sway-referenced support. SOT condition 5 = eyes closed, sway-referenced support. SOT condition 6 = sway-referenced vision, sway-referenced support.
